# Association of ileocolic pedicle division with postoperative complications after restorative proctocolectomy and ileal pouch-anal anastomosis for ulcerative colitis

**DOI:** 10.1186/s12893-021-01428-4

**Published:** 2021-12-18

**Authors:** Emmanouil Tzatzarakis, Florian Herrle, Wolfgang Reindl, Nora Altmayer, Dominik Minas, Peter Kienle, Christoph Reissfelder, Flavius Şandra-Petrescu

**Affiliations:** 1 Department of Surgery, Universitätsmedizin Mannheim, Medical Faculty Mannheim, Heidelberg University, Theodor-Kutzer-Ufer 1-3, 68167 Mannheim, Germany; 2 Department of Medicine II, Universitätsmedizin Mannheim, Medical Faculty Mannheim, Heidelberg University, Mannheim, Germany; 3 Department of Surgery, Theresienkrankenhaus, Mannheim, Germany

**Keywords:** Inflammatory bowel disease, Anastomotic leak, Minimal-invasive surgery, Ileal pouch-anal anastomosis, Ileocolic pedicle division, Laparoscopy

## Abstract

**Background:**

When performing a restorative proctocolectomy (RPC) with an ileal pouch-anal anastomosis (IPAA), it is common practice to divide the ileocolic artery (ICA) if the patient has a tumor or dysplasia, or in order to gain sufficient length to secure a tension-free anastomosis. However, it is unclear whether there is an association between division of the ICA and the rate of postoperative complications.

**Methods:**

We retrospectively analysed all patients with ulcerative colitis who underwent RPC and IPAA in our department between January 2010 and December 2016. These were divided in two groups, with regard to the ICA being preserved (PRE group) or divided (DIV group). Complications such as stenosis or leakage of the IPAA, perianal fistulas, abscess formation within the lesser pelvis and pouchitis were analysed and compared between both groups.

**Results:**

We identified 130 patients meeting the study inclusion criteria, 49 patients in the PRE and 81 patients in the DIV group. No statistical significance was observed in IPAA leakages (p = 0.71), anastomotic strictures (p = 0.33), fistulas (p = 0.19) and pouchitis (p = 0.72). Abscess formation frequency was similar in both groups (p > 0.99). Moreover, short-term (p = 0.53) and long-term complications (p = 0.11) were similar in both groups. A higher conversion rate was observed in obese (p = 0.006) and male (p = 0.02) patients. Within the entire study population, fistulas and IPAA leakages were associated with a higher rate of anastomotic strictures (p = 0.008 and p = 0.02 respectively).

**Conclusion:**

Our data suggest similar IPAA related complications after either division or preservation of the ICA. Further trials are required in order to examine the trends observed in this study.

## Background

Restorative proctocolectomy with ileal pouch-anal anastomosis (IPAA) is the procedure of choice for patients with ulcerative colitis (UC), refractory to pharmacological treatment. J-Pouch is the most commonly used method for pouch formation. It is faster, easier to perform and it has a similar rate of complications in comparison with other types of pouch [1, 2]. In order to minimise the risk of anastomotic complications, one of the most important issues when performing an IPAA is to ensure a tension-free connection to the anus while preserving an optimal blood supply [3–5]. The mesenteric length is usually assessed intraoperatively by using the base of the symphysis pubis as a landmark, although some surgeons prefer to test whether the pouch reaches the anus itself [6]. If additional mesenteric length is required, several techniques are routinely used for that purpose. The most common are either the high division of the superior mesenteric pedicle (SMP) whilst preserving the ileocolic pedicle, or -vice-versa- the division of the latter while preserving the former [7, 8]. Other procedures involve preservation of the marginal vascular arcade (MVA) of the right hemicolon, which in turn allows for ligation of more mesenteric vessels [9], or peritoneal incisions along the SMP [10]. Division of the ileocolic artery (ICA) is primarily performed in several centres and is mandatory in case of oncological resection [11, 12]. However, the impact of this “sacrifice” on the rate of postoperative pouch-related complications is not fully understood. Similar to resection of the sigmoid colon with division of the inferior mesenteric artery (IMA), division of the ileocolic pedicle may influence morbidity due to pouch related complications. As shown by two prospective randomized studies on patients undergoing left hemicolectomy for benign disease, the rate of anastomotic leakage or defecatory disorders could be reduced by preserving the IMA [13, 14]. Likewise, a better blood supply of the ileum through preservation of the ICA may lead to a reduction in the rate of complications in patients with UC undergoing RPC and IPAA.

In our department, the ileocolic pedicle is primarily preserved in case of benign disease. This allows for more options at the time of pouch formation, should the length of the mesentery prove to be inadequate. Furthermore, peritoneal incisions along the SMP are routinely performed.

Since most of the patients with UC are young adults, a proper long-term function of the pouch represents a pivotal outcome parameter. To that end, the best possible surgical outcome is of high importance. This study aims to compare the pouch-related complications depending on preservation of the ileocolic pedicle, in patients with UC undergoing RPC.

## Materials and methods

A systematic literature research was performed in order to identify studies investigating the association of ICA division with the rate of pouch-related complications after IPAA. Search strategies included combinations of MeSH terms and text words related to “ulcerative colitis”, “colonic pouches”, “colectomy”, “postoperative complications”, “ileocolic artery” and “mesenteric lengthening”. After duplicates were excluded, all publications were analysed by abstract. Papers matching the search criteria were analysed by full manuscript and the relevant ones were listed in this manuscript.

Clinical records of patients undergoing restorative proctocolectomy and IPAA for ulcerative colitis between January 2010 and December 2016 in our department were retrospectively analysed regarding the pouch-related complications according to preservation or division of the ileocolic artery (ICA). Patients were divided into two groups, the ICA preservation- (PRE) and the ICA division-group (DIV). ICA was routinely preserved in our department. Reasons for ICA division were either malignancy, requiring an oncological resection with primary division, or lack of sufficient mesenteric length in order to ensure a tension-free anastomosis. Surgery was performed as previously described [15]. Both 2- and 3-stage restorative proctocolectomies were included in the study, and a J-shaped pouch was always created. Both hand-sewn and stapler pouch-anal anastomosis were performed. If too much tension on the IPAA was expected, as evaluated on-site by the surgeon using symphysis pubis as a landmark, several mesenteric lengthening procedures were applied (e.g., division of the last inferior mesenteric branches or peritoneal incisions along the SMP). The ICA was divided only as a last resort.

Pouch-related complications such as IPAA stenosis (defined as clinically relevant stenosis requiring a therapeutic intervention such as dilation using Hegar dilators or manual dilatation and bougienage of the anus or surgical revision), leakage and fistulas, abscess formation in the lesser pelvis and pouchitis were analysed in both groups. Anastomotic leakage was defined as a visible defect of the colonic wall at the level of the anastomosis, enabling a communication between the intra- and extraluminal space, as assessed by postoperative sigmoidoscopy. We further divided complications into short- (anastomotic leakage, abscess formation) and long-term ones (anastomotic strictures, fistula formation).

Patients underwent pouchoscopy routinely after 4 and/or 12 weeks following IPAA. Afterwards, there was a follow-up yearly or upon appearance of symptoms. The findings over a period of two years were extracted from our endoscopic database and were analysed. No missing data was reported.

The study was reviewed and approved by the Medical Ethics Commission II of the Medical Faculty Mannheim, Heidelberg University (Votum-Nr. 2021-820). Given its retrospective nature, an informed consent of the patients participating in it was waived. All research methods were performed in accordance with the Declaration of Helsinki.

Quantitative variables are presented by median value and range. In order to compare the two groups regarding relative frequencies, Chi-squared test or Fisher’s exact test were performed, as deemed appropriate. The result of the statistical test was considered as significant for p < 0.05. Statistical calculations were done using SAS software, release 9.3 (SAS Institute Inc., NC, USA).

## Results

After elimination of duplicates, 984 results were identified within the literature search. All abstracts were analysed and 71 articles were reviewed in detail and assessed for eligibility. Of those, 14 articles were considered relevant to the topic of the study. No study on the influence of ICA division on the postoperative outcome after ileo-anal pouch (IAP) formation could be identified (Table 1). ICA and SMP division were identified as the most used procedures to ensure enough mesenteric length.

**Table 1:** Relevant studies: results of the literature search

Author	Year	No. of patients	Complications investigated	Lengthening techniques	Influence of ICA division on postoperative complications
Araki et al. [12]	2006	220	+	ICA (−), SMP (−), MVA (+)	n.i
Farouk et al. [25]	1998	1508	+	−	n.i
Fazio et al. [21]	2013	3703	+	−	n.i
Goes et al. [9]	1995	6*	−	MVA (+)	n.i
Ismail et al. [10]	2018	25*	−	SLI, SMP (−), ICA (−)	n.i
Klos et al. [26]	2014	178	+	−	n.i
Martel et al. [7]	1998	65	+	SMP (−)	n.i
Martel et al. [27]	2002	12*	−	ICA (−), SMP (−)	n.i
Meagher et al. [23]	1998	1310	+	−	n.i
Rickard et al. [28]	2007	516	+	−	n.i
Thirlby et al. [11]	1995	74	−	ICA (−), SMP (−)	n.i
Uchino et al. [29]	2018	2376	+	−	n.i
Utsunomiya et al. [8]	1980	13	+	−	n.i
Wu et al. [20]	2014	134	+	SLI, ICA, MEI	n.i

*ICA* ileocolic artery, *SMP* superior mesenteric pedicle, *MVA* marginal vascular arcade, *SLI* “stepladder” incisions, *MEI* mesentery incisions, (+) preservation, (−) division, *n.i.* not investigated

*The study was performed on cadavers

In our study, 130 patients, 58 females (44.6%) and 72 males (55.4%), met the inclusion criteria (Table 2). Of those, 49 were included in the PRE and 81 in the DIV group. All operations were performed by a single surgeon. Most procedures were performed laparoscopically (PRE 87.8%, DIV 77.8%) with a higher conversion rate in the DIV group (2.0% vs. 14.8%). Conversion rate was significantly associated with a body-mass index (BMI) > 30 kg/m^2^ (p = 0.006) as well with male sex (p = 0.002). Reason for converting was tumor size in the single patient in the PRE group, whereas in the DIV group three patients had a large tumor, two showed excessive bleeding and seven were obese [with a short mesentery (n = 4), adhesions due to previous operations (n = 2) or high ventilating pressure during laparoscopy (n = 1)]. No statistical differences were found regarding BMI and type of procedure (2- or 3-stage, p = 0.33) or division of the ICA (p > 0.99). There was a tendency towards more 3-stage procedures in men (p = 0.11). IPAA was performed transanally, using a circular stapler in all of the PRE and in 79.0% of the DIV group patients. All patients with a BMI > 30 kg/m^2^ received a stapled anastomosis (p = 0.07). Hand-sewn anastomosis was performed in the remaining 21.0% of the DIV group patients. The frequency of control pouchoscopies performed after IPAA was similar in both groups, with a mean of 3.4.

**Table 2:** Baseline population characteristics

	PRE (n = 49)	DIV (n = 81)	Total (n = 130)	p value
Median age (years), [IQR]	38 [27–53]	43 [33–51]	42 [31–51]	
Sex (male/female)	26/23	46/35	72/58	0.71
BMI (kg/m^2^)				
< 30	37 (75.5%)	65 (80.2%)	102 (78.5%)	> 0.99
≥ 30	8 (16.3%)	13 (16.1%)	21 (16.2%)	
No data	4 (8.2%)	3 (3.7%)	7 (5.4%)	
Procedure				
Laparoscopy	43 (87.8%)	63 (77.8%)	106 (81.5%)	0.16
Laparotomy	5 (10.2%)	6 (7.4%)	11 (8.5%)	0.75
Conversion	1 (2.0%)	12 (14.8%)	13 (10.0%)	0.03
Two-stage	31 (63.3%)	46 (56.8%)	77 (59.2%)	0.47
Three-stage	18 (36.7%)	35 (43.2%)	53 (40.8%)	0.47
Anastomosis				
Stapler	49 (100%)	64 (79.0%)	113 (86.9%)	< 0.001
Hand-Sewn	0 (0.0%)	17 (21.0%)	17 (13.1%)	
Frequency of postoperative endoscopy (mean)	3.39	3.40	3.40	> 0.99

*PRE* ileocolic artery preserved, *DIV* ileocolic artery divided, *BMI* body-mass-index, *IQR* interquartile range

The pouch-related complications, which occurred during the hospitalisation period or during the follow-up period, were analysed in both groups (Table 3). Overall complication rate was similar in both groups (p > 0.99). Clinically relevant stenosis of the J-Pouch, perianal fistulas and the rate of anastomotic leakage were not statistically different between the two groups (p = 0.33, 0.19 and 0.71 respectively). In the entire cohort, half of the patients with fistula formation and 60% of those with anastomotic leakage consequently developed an anastomotic stenosis (p = 0.008 and p = 0.02 respectively). There was no significant association between abscess formation and anastomotic stenosis (p = 0.30).

**Table 3:** Rate of complications

	PRE (n = 49)	DIV (n = 81)	Total (n = 130)	p value
Patients with any complication*	22 (44.9%)	36 (44.4%)	58 (44.6%)	> 0.99
Long-term	10 (20.4%)	8 (9.9%)	18 (13.8%)	0.11
Stenosis of the J-Pouch	6 (12.2%)	5 (6.2%)	11 (8.5%)	0.33
Perianal fistulas	4 (8.2%)	2 (2.5%)	6 (4.6%)	0.19
Short-term	3 (6.1%)	9 (11.1%)	12 (9.2%)	0.53
Anastomotic leakage	2 (4.1%)	6 (7.4%)	8 (6.2%)	0.71
Abscess in the lesser pelvis	1 (2.0%)	3 (3.7%)	4 (3.1%)	> 0.99
Pouchitis	22 (44.9%)	33 (40.7%)	55 (42.3%)	0.72

*PRE* ileocolic artery preserved, *DIV* ileocolic artery divided

*More than one complications possible per patient

Both groups developed similarly frequent abscesses in the lesser pelvis (p > 0.99). With regard to the anastomotic method, stapler vs. hand-sewn, no association with the rate of stenosis or leakage of the IPAA was observed (p > 0.99). Furthermore, the endoscopic findings identified no case of enteric ischemia. Moreover, the analysis of the frequency of pouchitis found no significant difference between the groups (p = 0.72) (Table 3). Short-term complications were similar in both groups (p = 0.53), whereas long-term ones tended to be more common in the PRE group (p = 0.11).

The number of patients requiring an ostomy formation was similar in both groups (p = 0.36). No patient required formation of a new pouch. One patient in the PRE group underwent pouch extirpation due to a persistent fistula. The need for endoluminal vacuum therapy and/or CT guided drainage due to abscess formation in the lesser pelvis were also similar in both groups (p > 0.99) (Table 4).

**Table 4:** Frequency of postoperative interventions

	PRE (n = 49)	DIV (n = 81)	p value
New pouch	0 (0.0%)	0 (0.0%)	> 0.99
Ostomy with pouch preservation	2 (4.1%)	2 (2.5%)	0.63
Ostomy with pouch extirpation	1 (2.0%)	0 (0.0%)	0.38
Endo-VAC with protective ostomy	1 (2.0%)	1 (1.2%)	> 0.99
CT guided drainage of an abscess	1 (2.0%)	1 (1.2%)	> 0.99
Endo-washer	0 (0.0%)	1 (1.2%)	> 0.99
Laser and argon beamer coagulation (cuffitis)	1 (2.0%)	0 (0.0%)	0.38

*PRE* ileocolic artery preserved, *DIV* ileocolic artery divided, *Endo-VAC* endoluminal vacuum-assisted closure, *CT* computer tomography

## Discussion

A tension-free IPAA is of major importance for an optimal surgical outcome and thus a better quality of life in patients with UC. To our knowledge, the present study is the first to investigate the association of ICA-division with postoperative morbidity after RPC and IPAA. Several authors investigate ICA-division as a means of gaining mesenteric length; however, none of them studied its association with postoperative complications (Table 1).

The overall postoperative complication rate in our study was 44.6%. Those included complications presenting within the postoperative period, as well as during control visits afterwards. Short-term complications, i.e., anastomotic leakage and abscess formation, were found similar in both groups. The overall incidence of IPAA leakage was 6.2%, which was similar to the literature (5–18%) [16, 17]. Abscess formation within the small pelvis had a frequency comparable to the literature (3.1% vs up to 19%) [18, 19].

On the contrary, we observed a trend towards more long-term complications, i.e., anastomotic stricture and fistula formation, in the PRE group (Table 3). Although not statistically significant, patients in the PRE group developed numerically more clinically relevant strictures of the IPAA than in the DIV (12.2% vs. 6.2%), at a similar rate with previously reported frequencies of 10 to 16% [20–22]. Performing a stapled or a hand-sewn IPAA did not correlate with the frequency of clinically relevant strictures within the DIV group. Similarly, the rate of pouch related fistulas did not reach a significant difference between the groups. Our results correlate with those of a systematic review of 28 studies investigating the outcomes of colectomy and IPAA in patients with UC, which reports a fistula rate of 6–8% [18]. Fistula formation as well as anastomotic leakage were found to be a significant prognostic factor for the development of anastomotic strictures and, subsequently, clinically relevant stenosis, probably due to the ongoing healing process and scar formation. Abscesses, on the other hand, were not significantly associated with a later appearance of clinically relevant stenosis.

A common complication after IPAA is inflammation of the pouch mucosa itself, defined as pouchitis. The overall risk of pouchitis after RPC with IPAA was described in the literature to be 18% after one year, climbing up to 48% within 10 years [23]. Recent studies show an incidence of 44% within the first 10 years or up to 70% within a 20-year period [22, 24]. In our study, ICA division did not significantly correlate with the rate of pouchitis. Pouch failure, defined as excision of the pouch or maintaining of a permanent ileostomy, occurred only in one patient in the PRE group, being lower than the bibliographic findings of 5–10% [24].

We observed a significantly higher conversion rate in the DIV group. Reasons for conversion reported in the patients’ records were obesity, specimen size (all of which were cases of malignancy) or excessive bleeding. Among the patients with a BMI > 30 kg/m^2^, a short mesentery was often described; this was usually observed in terms of a three-stage procedure, suggesting that the previous operations led to more abdominal adhesions and a contracted mesentery. Furthermore, most of those who underwent conversion were men. This portrays the fact, that a tension-free IPAA formation is often difficult in case of obesity, manly pelvis, and/or previous operations, which in turn makes ICA division essential, in order to gain sufficient mesenteric length.

As mentioned above, ICA preservation was always attempted in our department. With the exception of oncologic resections, ICA was routinely divided only when supplementary mesenteric length was needed. This represents a form of selection bias in the DIV group.

The present study found no significant differences between the two groups, suggesting that the preservation or division of the ICA does not correlate with postoperative complications after RPC. However, looking at the absolute numbers, a trend towards more long-term complications in the PRE group is suggested. The main limitations of this study are its non-randomized and retrospective nature. Therefore, the conclusions drawn here are limited. However, all operations were performed by a single surgeon, which ensures a consistency regarding the technical aspects and the decision-making during the operation. Thus, our data could serve to stimulate further scientific research towards prospective clinical trials on the association of ICA division with the IPAA related complications.

## Conclusion

In our study, ICA division or preservation had no significant association with IPAA related complications after RPC, suggesting that it can be divided whenever necessary, so as to achieve a tension-free anastomosis. However, ICA preservation may be primary attempted, in order to ensure the availability of more mesenteric lengthening options at the time of pouch formation.

## Data Availability

The datasets used and/or analysed during the current study are available from the corresponding author on reasonable request.

## Abbreviations

BMI: Body-mass index
CT: Computer tomography
DIV: Division group
IAP: Ileo-anal pouch
ICA: Ileocolic artery
IMA: Inferior mesenteric artery
IPAA: Ileal pouch-anal anastomosis
MEI: Mesenteric incisions
MVA: Marginal vascular arcade
n. i.: Not investigated
PRE: Preservation group
RPC: Restorative proctocolectomy
SLI: Stepladder incisions
SMP: Superior mesenteric pedicle
UC: Ulcerative colitis
VAC: Vacuum-assisted closure
